# Effects of red blood cell transfusion on patients undergoing cardiac surgery in Queensland – a retrospective cohort study

**DOI:** 10.1186/s13019-024-02974-7

**Published:** 2024-08-01

**Authors:** Nchafatso. G. Obonyo, Vikash Dhanapathy, Nicole White, Declan P. Sela, Reema H. Rachakonda, Matthew Tunbridge, Beatrice Sim, Derek Teo, Zohaib Nadeem, Louise E. See Hoe, Gianluigi Li Bassi, Jonathon P. Fanning, John-Paul Tung, Jacky Y. Suen, John F. Fraser

**Affiliations:** 1 Critical Care Research Group, The Prince Charles Hospital, Brisbane, QLD Australia; 2 Institute of Molecular Bioscience, The University of Queensland, Brisbane, QLD Australia; 3 Initiative to Develop African Research Leaders (IDeAL), KEMRI-Wellcome Trust Research Programme, Kilifi, Kenya; 4 Wellcome Trust Centre for Global Health Research, Imperial College London, London, UK; 5 Australian Centre for Health Services Innovation and Centre for Healthcare Transformation, School of Public Health and Social Work, Queensland University of Technology, Brisbane, QLD Australia; 6 School of Pharmacy and Medical Sciences, Griffith University, Gold Coast, QLD Australia; 7 Nuffield Department of Population Health, University of Oxford, Oxford, UK; 8 Wesley Medical Research, The Wesley Foundation, Auchenflower, Brisbane, QLD Australia; 9 Intensive Care Unit, St Andrew’s War Memorial Hospital, Spring Hill, Brisbane, QLD Australia; 10 Intensive Care Unit, The Wesley Hospital, Auchenflower, Brisbane, QLD Australia; 11 Clinical Services and Research, Australian Red Cross Lifeblood, Brisbane, QLD Australia; 12 Faculty of Health, Queensland University of Technology, Brisbane, QLD Australia; 13 School of Medicine, Griffith University, Gold Coast, QLD Australia; 14 Division of Cardiac Surgery, Department of Surgery, Johns Hopkins School of Medicine, Baltimore, MD USA

**Keywords:** Red blood cell transfusion, Cardiac surgery, Age of packed red blood cells, Storage lesion

## Abstract

**Background:**

Packed red blood cell (pRBC) transfusion is a relatively safe and mainstay treatment commonly used in cardiac surgical patients. However, there is limited evidence on clinical effects of transfusing blood nearing end-of shelf life that has undergone biochemical changes during storage.

**Objective:**

To investigate evidence of associations between morbidity/mortality and transfusion of blood near end of shelf-life (> 35 days) in cardiac surgical patients.

**Methods:**

Data from the Queensland Health Admitted Patient Data Collection database 2007–2013 was retrospectively analysed. Coronary artery bypass graft and valvular repair patients were included. Multivariable logistic regression was used to examine the effect of pRBC age (< 35 days vs. ≥ 35 days) on in-hospital mortality and morbidity. As secondary analysis, outcomes associated with the number of pRBC units transfused (≤ 4 units vs. ≥ 5 units) were also assessed.

**Results:**

A total of 4514 cardiac surgery patients received pRBC transfusion. Of these, 292 (6.5%) received pRBCs ≥ 35 days. No difference in in-hospital mortality or frequency of complications was observed. Transfusion of ≥ 5 units of pRBCs compared to the ≤ 4 units was associated with higher rates of in-hospital mortality (5.6% vs. 1.3%), acute renal failure (17.6% vs. 8%), infection (10% vs. 3.4%), and acute myocardial infarction (9.2% vs. 4.3%). Infection carried an odds ratio of 1.37 between groups (CI = 0.9–2.09; *p* = 0.14) and stroke/neurological complications, 1.59 (CI = 0.96–2.63; *p* = 0.07).

**Conclusion:**

In cardiac surgery patients, transfusion of pRBCs closer to end of shelf-life was not shown to be associated with significantly increased mortality or morbidity. Dose-dependent differences in adverse outcomes (particularly where units transfused were > 4) were supported.

**Supplementary Information:**

The online version contains supplementary material available at 10.1186/s13019-024-02974-7.

## Background

Cardiac surgery patients are among the highest consumers of allogenic packed red blood cells (pRBCs) transfusion in hospitals. Daly et al., found a transfusion rate of 57% of all cardiac surgery patients in Australia, while in the US, it is estimated that cardiac surgery alone accounts for 20% of all pRBCs transfused [1, 2]. Reasons for such extensive use in the cardiac surgical setting include higher rates of perioperative blood loss and anaemia, as well as the platelet destruction and haemodilution effects of cardiopulmonary bypass [3].

Indications for pRBC transfusion in cardiac surgery patients are complex – given the specific demographics and pathophysiological processes in this population, transfusion requirements are considered to be distinct from other patient groups. Factors complicating the use of blood transfusion include underlying compromised coronary artery circulation and/or cardiac output, high volume intraoperative blood loss, and post-operative bleeding/coagulopathy consequent to cardiopulmonary bypass, as well as the use of anticoagulation [4]. The National Blood Authority (NBA) in Australia published guidelines on the use of perioperative blood transfusion, making particular suggestions for their use in cardiac surgery [5]. In summary, it is recommended that patients should not receive pRBC transfusion when haemoglobin is > 100 g/L but it may be appropriate in patients with haemoglobin > 80 g/L, in the absence of acute myocardial infarction or cerebrovascular ischaemia [5]. A meta-analysis on restrictive versus liberal pRBC transfusion thresholds (studying 31 randomised controlled trials) concluded that a restrictive transfusion threshold of 80 g/L in patients undergoing cardiac surgery was strongly recommended [6].

It has been widely demonstrated that perioperative anaemia is an independent risk factor for all-cause mortality and morbidity – for instance, there is a 2- to 3-fold increase in risk of post-operative stroke and acute renal failure [2, 3]. Further, it is a well-established phenomenon that significant anaemia causes systemic vasodilation and consequent decrease in peripheral vascular resistance, potentially necessitating the use of higher amounts of vasopressors [7]. However, addressing anaemia and fluid loss with pRBCs is not without pertinent risks. Allogenic pRBC transfusion is known to confer immunosuppression and microcirculatory compromise – particular complications noted in cardiac surgery patients include infection (nosocomial pneumonia and sternal wound infection), atrial fibrillation, myocardial infarction, and acute renal failure among others [7, 8]. Evidence consistently demonstrates increased mortality in patients undergoing cardiac surgery who receive pRBC transfusion [7].

Stone et al., demonstrated a dose-dependent relationship between the number of RBC units transfused and mortality after cardiac surgery with 4 or more units being a strong independent risk factor for 30-day mortality [9]. Koch et al., similarly, found a dose-dependent increase in mortality with every unit after 4 units and concluded than for patients who underwent coronary artery bypass grafting (CABG) and received less than 4 units transfusion, observed mortality was 1% [8]. As such, when RBC transfusion is indicated, the NBA stipulates single unit transfusions with re-assessment of clinical status before additional units are given [5]. It should be noted, however, that the aetiology of death in these patients remains unclear [7]. Studies evaluating mortality and complications of pRBCs in cardiac surgery patients have mostly focussed on isolated CABG surgeries – the limited data available on isolated valvular repair surgeries has failed to show any mortality difference with the use of pRBCs [7, 10].

Transfusion-related morbidity/mortality has been attributed to the storage of pRBCs, as stored pRBCs undergo various biochemical changes collectively known as ‘storage lesions’. These changes are believed to promote immunosuppression, impaired oxygen unloading, and systemically compromised microcirculation among other problems [3, 7, 11–13]. These changes are known to occur within 2 weeks of storage [7]. While it has been shown that storage of pRBCs for longer than 14 days leads to more extensive, irreversible storage changes (compared to pRBCs fresher than 14 days where most lesions are reversible), the current evidence is undetermined on whether longer storage translates to a clinical difference in adverse effects [14].

The Red-Cell Storage Duration Study (RECESS trial) was a large, multicentre RCT comparing cardiac surgery patients receiving fresh (10 days or less) versus old blood (21 days or more) which showed no association between age of pRBCs and mortality or other adverse outcomes [12]. This finding was echoed by two similar multicentre RCTs investigating age of pRBC transfusions in the general hospital population – that is, the Age of Blood Evaluation (ABLE) trial and the Informing Fresh versus Old Red Cell Management (INFORM) trial – as well as in the critically ill population (Standard Issue Transfusion versus Fresher Red-Cell Use in Intensive Care (TRANSFUSE) trial) [4]. As with the ABLE and INFORM trials, the RECESS trial did not adequately include pRBCs stored longer than 35 days given the scarcity of such blood in practice [4, 12]. Ultimately, in 2016, Goel et al., showed increased adverse outcomes associated with the use of pRBCs within the last 7 days of their 42-day storage limit in high-risk medical patients [15]. As such, we investigated the hypothesis that there is an association between morbidity/mortality and transfusion of blood near end of shelf-life, particularly in the cardiac surgery population.

### Aims

This study aimed to evaluate the effects of blood transfusion on mortality and morbidity with pRBCs close to end of shelf-life (≥ 35 days) versus fresher blood on patients undergoing cardiac surgery through a multi-centre retrospective database analysis. As a parallel objective, transfusion-related adverse outcomes in relation to quantity of pRBCs transfused were characterised.

## Methods

### Setting

This was a retrospective cohort study on all cardiac surgery patients receiving blood transfusions in Queensland public hospitals between 2007 and 2013 conducted in accordance with the STROBE guideline (Appendix 1). Queensland Health hosts four cardiothoracic centres in the state, namely The Prince Charles Hospital, The Princess Alexandra Hospital, Gold Coast University Hospital, and Townsville University Hospital. Combined, these centres perform approximately 2600 adult cardiac surgeries each year [16].

### Study population

**Fig. 1:**
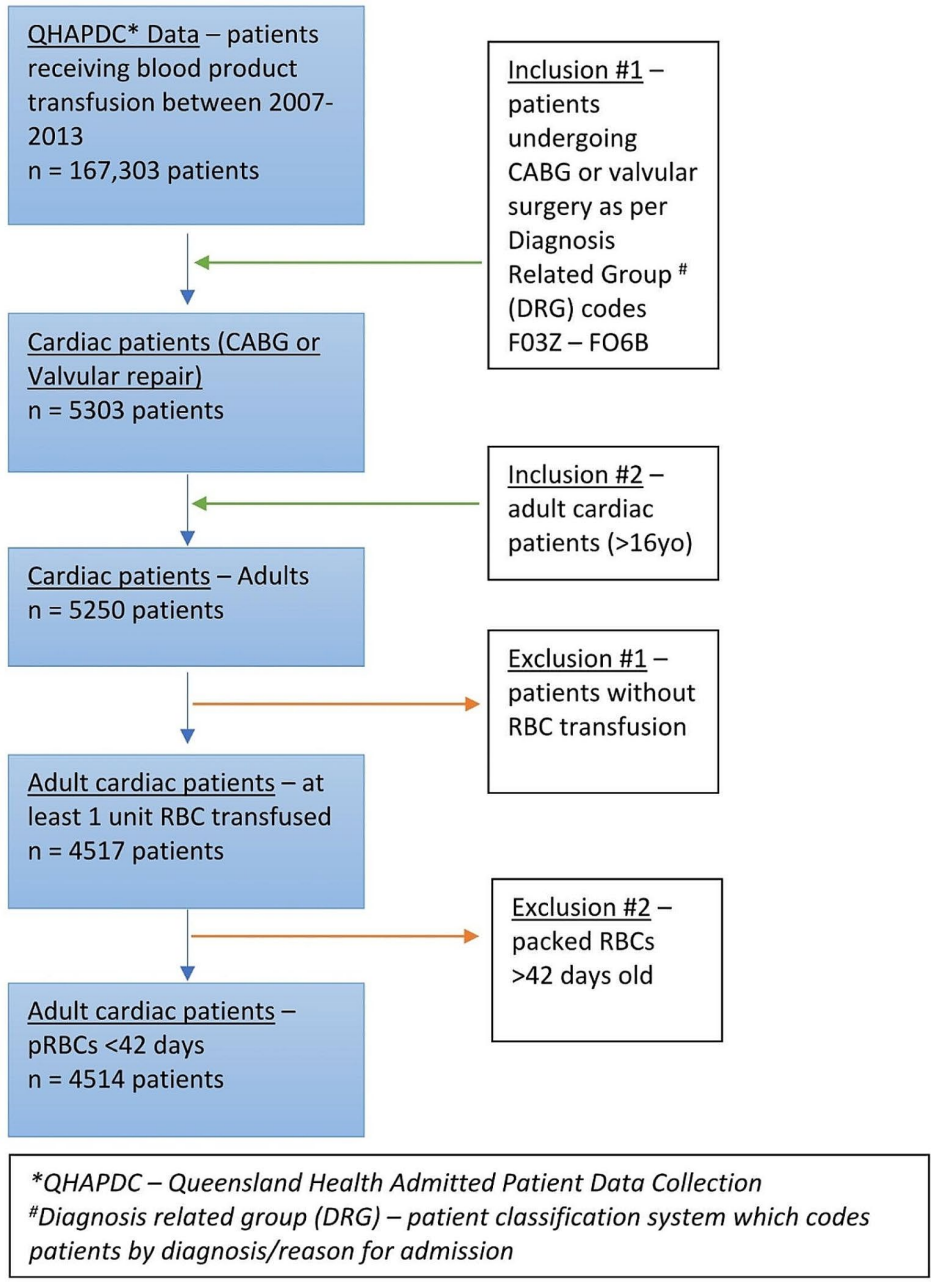
Patient inclusion and exclusion characteristics

Patients were eligible for inclusion if they were 16 years or older and underwent CABG surgery or Valvular repair/replacement surgery (Fig. 1).

Patients were excluded if they did not receive at least one unit of pRBC transfusion. Since the maximum allowed shelf-life of pRBCs in Australia is 42 days, any entries of pRBCs beyond this age were taken as errors in data entry and excluded from analysis.

### Data collection

Patient transfusion data is managed centrally on AUSLAB, the laboratory information system of Pathology Queensland, a state government service which catalogues every patient receiving blood product transfusions in a Queensland public hospital. Information associated with the transfusion such as blood product type, age of blood product and quantitative also collected.

AUSLAB data was crossmatched with the Queensland Health Admitted Patient Data Collection (QHAPDC) database, a state-wide data collection service capturing information on every patient admitted to a Queensland Health public hospital. Routinely collected information available from QHAPCE includes admission outcome, hospital length of stay, and attributed International Classification of Diseases (ICD) codes for primary and additional diagnoses.

### Study outcomes

The primary outcome was in-hospital mortality. Secondary outcomes were the incidence of transfusion-associated complications. Complications of interest were infection, atrial fibrillation, other dysrhythmia, cardiac arrest/acute myocardial infarction, acute renal failure, and stroke/neurological complications. Complications during hospital admission were identified for each patient using ICD codes recorded in QHAPDC. A list of ICD-codes for each complication is provided in Appendix 2.

### Data analysis

The primary patient groups compared were those patients receiving pRBCs with an average age of < 35 days versus pRBCs with an average age ≥ 35 days. As a secondary analysis, patients receiving ≤ 4 units pRBCs were compared to those receiving ≥ 5 units. This was based on previously published data from the Australian and New Zealand Society for Cardiac and Thoracic Surgery (ANZSCTS) Cardiac Surgery Database reported by McQuilten et al., 2014 [17]. Cohort characteristics and outcomes were first summarised descriptively, using medians with interquartile ranges for continuous variables, and frequencies with percentages for categorical variables. Continuous outcomes (i.e., hospital length of stay) were summarised using linear regression. Categorical outcomes of in-hospital mortality and frequency of complications (specifically, infection, atrial fibrillation, other dysrhythmia, cardiac arrest/AMI, acute renal failure, cerebrovascular events) were summarised using multivariable logistic regression to determine odds ratio of incidence. Variables used as covariates in this analysis, for both outcomes of mortality and complication incidence, included: Age, sex, year of transfusion, HLOS, surgery indication, number of blood products (RBC, plasma, platelet, cryoprecipitate, granulocyte), and incidence complications (Infection, AF, Other Dysrhythmia, Cardiac arrest/AMI, Acute renal failure, Stroke/neurological).

Statistical significance for mortality and complication incidence was defined as *p* < 0.05. Data was analysed using STATA Version 17.

## Results

A total of 4 514 eligible patients undergoing cardiac surgery and receiving blood transfusion were observed in this study (Fig. 1). Characteristics of the included patients are summarised in Table 1. Patients were predominantly male (61.5%) and over the age of 60 (75.1%). CABG procedures accounted for 55.8% of indication for surgery, the rest being valvular repair/replacement surgery. Most patients were transfused 4 units or less of pRBCs (73.5%); in terms of auxiliary blood products, 35.7% of patients required platelets while 32.2% required fresh frozen plasma. 86.8% of patients were discharged home and 2.4% of patients died in hospital.

**Table 1:** Demographics and characteristics of patients

Characteristic	*N*, 4514 (%)
**Sex**	
Male	2778 (61.5)
Female	1736 (38.5)
**Age group**	
16–30	77 (1.7)
31–40	109 (2.4)
41–50	272 (6.0)
51–60	669 (14.8)
61–70	1249 (27.7)
71–80	1621 (35.9)
80+	517 (11.5)
**Surgery type**	
Coronary Artery Bypass Graft (CABG)	2517 (55.8)
Valvular repair	1997 (44.2)
**No. pRBC units**	
<=4	3319 (73.5)
>=5	1195 (26.5)
**pRBC age**	
<=7 days	218 (4.8)
8–21 days	2665 (59)
22–27 days	821 (18.2)
28–34 days	518 (11.5)
35–42 days	292 (6.5)
**Auxiliary blood products used**	
Whole blood	1 (0.02)
Plasma products	1454 (33.2)
Platelet products	1610 (35.7)
Cryoprecipitate	283 (6.2)
Granulocytes	17 (0.4)
**Hospital length of stay**	
<=7days	842 (18.7
8–14 days	2080 (46.1)
15–30 days	1250 (27.7)
30 + days	342 (7.6)
**Patient outcome**	
Died in hospital	108 (2.4)
Discharged home/other	4406 (97.6)

N = number of patients; pRBC = packed red blood cells

Of the total population, 4 222 (93.5%) patients received pRBCs with average age fresher than 35 days and 292 (6.5%) received pRBCs ≥ 35 days (Table 2). In-hospital mortality was 2.5% in the < 35-day cohort and 1% in the ≥ 35-day cohort, though this result was not statistically significant (OR 0.63; CI = 0.18 to 2.18; *p* = 0.46). No clinical or statistical differences in frequency of complications were observed between groups.

**Table 2:** Demographics and outcomes of patients receiving RBC products < 35 days old versus ≥ 35 days old

	Age of pRBC		
**Variable**	**< 35 days**	**≥ 35 days**	Odds ratio; (95% CI); p-value*
**Age (median, (IQR))**	70 (61,77)	70 (60.5,76)	-
**Sex**			
Females (n, %)	1618 (40.2)	118 (40.4)	-
**Cardiac surgical procedure**			
CABG^+^ (n, %)	2351 (55.7)	166 (56.9)	-
Valve surgery (n, %)	1871 (44.3)	126 (43.2)	
No. units pRBC (median, (IQR))^†^	3 (2,5)	2 (1,3)	-
Hospital Length of Stay (HLOS) (median, (IQR))	11 (8,18)	10 (8,16.5)	(OR=-1.3; CI=-2.54 – -0.06; *p* = 0.04)
**Outcome**			
Death (n, %)	105 (2.5)	3 (1)	(OR = 0.63; CI = 0.18–2.18; *p* = 0.46)
Discharged home (n, %)	4117 (97.5)	289 (99)	
**Complications**			
Infection (n, %)	220 (5.2)	14 (4.8)	(OR = 1.23; CI = 0.68–2.25; *p* = 0.5)
Atrial fibrillation (n, %)	1909 (45.2)	131 (44.9)	(OR = 1.02; CI = 0.79–1.31; *p* = 0.9)
Other dysrhythmia (n, %)	436 (10.3)	25 (8.6)	(OR = 0.88; CI = 0.57–1.37; *p* = 0.58)
Cardiac arrest/AMI (n, %)	239 (5.7)	12 (4.1)	(OR = 0.84; CI = 0.45–1.55; *p* = 0.45)
Acute renal failure (n, %)	452 (10.7)	25 (8.6)	(OR = 0.83; CI = 0.53–1.3; *p* = 0.42)
Stroke/neurological complications (n, %)	170 (4.03)	12 (4.1)	(OR = 1.02; CI = 0.55–1.88; *p* = 0.95)
Total (%)	4222 (93.5%)	292 (6.5%)	

Age = years; HLOS = days; pRBC = packed red blood cells; 95% CI = 95% confidence interval; IQR = interquartile range; CABG = coronary artery bypass graft; AMI = acute myocardial infarction

*Odds ratio, using multivariate logistic regression, calculated for outcomes of hospital length-of-stay, in-hospital mortality, complication incidence

Comparing patients who received ≤ 4 units of pRBCs (*n* = 3 319, 73.5%) to those who received ≥ 5 units (*n* = 1195, 26.5%), Table 3, the in-hospital mortality was 5.6% and 1.3%, respectively (OR 1.08), though this result was statistically non-significant (CI = 0.56 to 2.09; *p* = 0.81). Hospital length of stay was respectively longer (15 days versus 10 days median; Estimate = 6.02; CI = 5.36 to 6.69; *p* = 0.001)). The frequency of complications was higher for patients receiving ≥ 5 units of pRBCs including; acute renal failure (17.6% vs. 8%), infection (10% vs. 3.4%), and cardiac arrest/acute myocardial infarction (9.2% vs. 4.3%); *p* < 0.0011. Odds ratio for infection, comparing ≥ 5-unit to ≤ 4-unit groups, was 1.37 (CI = 0.9 to 2.09; *p* = 0.14) and 1.59 for stroke/neurological complications (CI = 0.96 to 2.63; *p* = 0.07) (Table 3). Table 4 shows comparison between patients undergoing coronary artery bypass grafting (CABG) and valve repair surgery.

**Table 3:** Demographics and outcomes in patients receiving ≤ 4 versus ≥ 5 units of pRBCs

	*No. units pRBC*		
**Variable**	**≤ 4 units**	**≥ 5 units**	Odds ratio; 95% CI; p-value*
**Age (median, (IQR))**	69 (60,76)	71 (62,77)	-
**Sex**			
Females (n, %)	1307 (39.4)	429 (35.9)	-
pRBC age (median, (IQR))	19 (14, 26)	17 (13, 24)	
**Cardiac surgical procedure**			
CABG^+^ (n, %)	1935 (58.3)	582 (48.7)	-
Valve surgery (n, %)	1384 (41.7)	613 (51.3)	
Hospital Length of Stay (HLOS) (median, (IQR))	10 (8,16)	15 (10,24)	(OR = 6.02; CI = 5.36–6.69; *p* = 0.001)
**Outcome**			
Death (n, %)	41 (1.3)	67 (5.6)	(OR = 1.08; CI = 0.56–2.09; *p* = 0.81)
Discharged home (n, %)	3278 (98.7)	1128 (94.4)	
**Complications**			
Infection (n, %)	114 (3.4)	120 (10)	(OR = 1.37; CI = 0.9–2.09; *p* = 0.14)
Atrial fibrillation (n, %)	1435 (43.2)	605 (50.6)	(OR = 1.09; CI = 0.88–1.36; *p* = 0.43)
Other dysrhythmia (n, %)	306 (9.2)	155 (13)	(OR = 0.88; CI = 0.64–1.21; *p* = 0.42)
Cardiac arrest/AMI (n, %)	141 (4.3)	110 (9.2)	(OR = 1.05; CI = 0.71–1.56; *p* = 0.8)
Acute renal failure (n, %)	267 (8)	210 (17.6)	(OR = 1.06; CI = 0.78–1.45; *p* = 0.7)
Stroke/neurological complications (n, %)	117 (3.5)	65 (5.4)	(OR = 1.59; CI = 0.96–2.63; *p* = 0.07)
Total (%)	3319 (73.5%)	1195 (26.5%)	

Age = years; pRBC age = days; HLOS = days; pRBC = packed red blood cells; 95% CI = 95% confidence interval; IQR = interquartile range; CABG = coronary artery bypass graft; AMI = acute myocardial infarction

*Odds ratio, using multivariate logistic regression, calculated for outcomes of hospital length-of-stay, in-hospital mortality, complication incidence

**Table 4:** Characteristics and outcomes in patients undergoing CABG vs. valve repair surgery

	Surgery type		
Variable	CABG	Valve repair	Odds ratio; 95% CI; *p*-value*
**Age (median, (IQR))**	69 (61,76)	71 (60,78)	
**Sex**			
Females (n, %)	856 (34)	880 (44.1)	
No. units pRBC (median, (IQR))	2 (2,4)	3 (2,5)	
pRBC age (median, (IQR))	19 (14, 25)	18 (13, 25)	
Hospital Length of Stay (HLOS) (median, (IQR))	11 (8,16)	12 (9,20)	(OR = 2.52; CI = 1.91–3.13; *p* = 0.001)
**Outcome**			
Death (n, %)	58 (2.3)	50 (2.5)	(OR = 0.91; CI = 0.55–1.5; *p* = 0.72)
Discharged home (n, %)	2459 (97.7)	1947 (97.5)	
**Complications**			
Infection (n, %)	77 (3.1)	157 (7.9)	(OR = 1.83; CI = 1.34–2.51; *p* = 0.001)
Atrial fibrillation (n, %)	1007 (40)	1033 (51.7)	(OR = 1.64; CI = 1.44–1.87; *p* = 0.001)
Other dysrhythmia (n, %)	252 (10)	209 (10.5)	(OR = 0.95; CI = 0.77–1.17; *p* = 0.62)
Cardiac arrest/AMI (n, %)	170 (6.8)	81 (4.1)	(OR = 0.51; CI = 0.38–0.69; *p* = 0.001)
Acute renal failure (n, %)	218 (8.7)	259 (13)	(OR = 1.34; CI = 1.09–1.66; *p* = 0.005)
Stroke/neurological complications (n, %)	102 (4.1)	80 (4)	(OR = 0.89; CI = 0.65–1.22; *p* = 0.47)
Total (%)	2517 (55.8)	1997 (44.2)	

Age = years; pRBC age = days; HLOS = days; pRBC = packed red blood cells; 95% CI = 95% confidence interval; IQR = interquartile range; CABG = coronary artery bypass graft; AMI = acute myocardial infarction

*Odds ratio, using multivariate logistic regression, calculated for outcomes of hospital length-of-stay, in-hospital mortality, complication incidence

## Discussion

In summary, it was found that transfusing pRBCs close to end of shelf-life (≥ 35 days) in cardiac surgery patients was not associated with increased early morbidity or mortality compared to patients receiving blood fresher than 35 days (Table 2). On comparing patients receiving ≤ 4 vs. ≥ 5 units of pRBC, higher rates of mortality and complications (specifically infection, acute renal failure and cardiac arrest/AMI) were observed in the ≥ 5 units group, though statistical significance in these results were not achieved (Table 3).

There is a lack of data studying pRBCs older than 35 days. Several observational studies including Yap et al., McKenny et al., and van de Watering et al., each demonstrated no association between pRBC storage age and poorer outcomes in cardiac surgery patients [18–20]. While Yap et al., specifically studied blood older than 30 days, McKenny et al., and van de Watering et al., excluded pRBCs older than 35 days. This study included pRBCs with age > 35 days (up to 42 days) and yet no difference in outcome was demonstrated.

Still, Koch et al., (in a larger study) found pRBCs older than 14 days to be a risk factor for mortality and renal failure; Andreasen et al., similarly found that pRBCs stored longer than 14 days were significantly associated with postoperative infection [21]. The results of these latter studies correlate with the fact that the majority of storage lesions develop by at least 2 weeks of storage [7]. Firstly, there is loss of 2,3-diphosphoglycerate (2,3-DPG), thought to be one of the principal biochemical changes in stored blood, which falls rapidly to undetectable levels within 2 weeks [22]. Similarly, irreversible morphological changes to stored pRBCs, associated with loss of function (such as altered corpuscle shape, deformability, aggregability, and osmotic fragility), occur by day 12 of storage [23, 24]. pH and lactic acid production plateau around 14 days while potassium concentration is beyond detectable levels at week 3 [25, 26]. As such, it may be drawn that the majority of clinically deleterious biochemical changes to stored pRBCs occur by the 14–21 day mark – further storage past this point, and certainly after 35 days, may be relatively minute and hence explain why no difference in mortality or morbidity was observed in this study with use of pRBCs older than 35 days (as well as those aforementioned studies where pRBCs older than 30 days were examined and, equally, no difference in mortality was evinced).

### Quantity pRBC units

Since a higher number of pRBC units are used per patient in the cardiac surgical setting, the association between the quantity of pRBCs transfused and adverse outcomes has been of particular interest. Koch et al., presented high-quality evidence demonstrating a strong dose-dependent relationship between pRBC transfusion and mortality, as well as all morbid outcomes studied (particularly postoperative infection, renal morbidity and cardiac morbidity), in patients undergoing isolated CABG surgery [8]. While each pRBC unit transfused was associated with an incremental increase in risk of adverse outcomes, risk of in-hospital mortality escalated rapidly after 5 units were given [8]. Similarly, subgroup analysis in the ACUITY trial of patients who underwent CABG showed that RBC transfusion was a strong, independent predictor of mortality, but only after ≥ 4 units [9]. Though statistical significance was not achieved, our study mirrored this inflection point, showing a higher rate of in-hospital mortality in the ≥ 5 units pRBC compared to those receiving < 4 units pRBC (5.6% vs. 1.2%, respectively) (Table 3). The lack of a statistically significant mortality difference in this study may be explained by the fact that patients included in our ≤ 4 units received at least one unit of pRBC, whereas the aforementioned studies included patients who received no pRBCs at all.

Besides mortality, Koch et al. have found the dose-dependent effects of pRBCs to hold true for postoperative infection, cardiac complications, renal failure and neurological complications [8]. Though statistical significance could not be achieved, rates of all co-morbid complications studied were notably higher in the ≥ 5 units group, with the highest absolute increases observed in infection, acute renal failure and cardiac arrest/AMI (parallel to Koch et al.) (Table 3). Infection occurred in 3.4% of patients in the ≤ 4 units group compared to 10% in the ≥ 5 units groups, with an odds ratio 1.37 (CI = 0.9–2.09; *p* = 0.14). The current literature maintains infection to be the foremost complication in cardiac surgery patients receiving blood transfusion [27, 28]. The most common infections observed in this setting include nosocomial pneumonia, followed by wound infections (sternal wound infection and saphenous vein graft site infection), and bacteraemia/sepsis [29, 30].

Further, the adverse outcome of stroke/neurological complications was found, in this study, to carry the largest odds ratio between the ≤ 4 units and ≥ 5 units groups (OR 1.59; CI = 0.96–2.63, *p* = 0.07), with incidence of 3.5% and 5.4%, respectively. This was not a statistically significant result, though we note this value to be comparable to Mariscalco et al., who demonstrated pRBC transfusion to be a strong dose-dependent determinant of stroke in the cardiac surgical population – transfusion of 4 units conferred an odds ratio of 1.82 for stroke/TIA compared to < 4 units [31–35].

### Limitations

This study has several limitations. Firstly, as a retrospective observational study, temporality is removed from the associations studied and the issue of reverse causation bias remains – as such, it is unclear whether less quantity of pRBCs resulted in healthier patients or healthier patients resulted in less pRBCs units used. Further, the increased generalisability obtained by analysing a multicentre population also sacrifices uniformity in blood transfusion practices across said hospitals. Neither state-wide nor departmental protocols for blood transfusion were identified. It should also be noted that during the data collection period of 2007–2013, Australia’s National Blood Authority published national patient blood-management guidelines for perioperative use (2012) with Irving et al., demonstrating a measurable reduction in perioperative blood transfusion in cardiac surgery across Australia (albeit, no differences in patient outcomes before and after 2012 were detected) [5]. What’s more, the data obtained through AUSLAB and QHAPDC registries, permitted maximal completeness of data and minimised data collection biases in sacrifice for detail – for example, data including patient comorbidities, haemoglobin levels, anticoagulation; units transfused in preoperative versus intraoperative/postoperative period, cardio-pulmonary bypass time; and specific outcome measures such as cause of death and ICU ventilation time/LOS. As such multivariate risk-adjustment or use of more robust clinical endpoints was not practicable with the data available to us. In addition, granularity of data to show the illness severity scores, as well as re-exploration for bleeding or tamponade was not available in the database analysed.

In conclusion, transfusion of pRBCs closer to end of shelf-life in cardiac surgical patients was not shown to be associated with significantly increased mortality or morbidity. However, there were dose-dependent differences in adverse outcomes in those receiving > 4 units of blood and blood products.

### Electronic supplementary material

Below is the link to the electronic supplementary material.

Supplementary Material 1

## Data Availability

Availability of data and materials: The datasets used and/or analysed during the current study are available from the corresponding author on reasonable request. Patient transfusion data is managed centrally on AUSLAB, the laboratory information system of Pathology Queensland, a state government service which catalogues every patient receiving blood product transfusions in a Queensland public hospital. AUSLAB data was crossmatched with the Queensland Health Admitted Patient Data Collection (QHAPDC) database, a state-wide data collection service capturing information on every patient admitted to a Queensland Health public hospital.

## Abbreviations

2-3-DPG: 2: 2,3-diphosphoglycerate
ABLE: Age of Blood Evaluation
ACUITY: Acute Catheterization and Urgent Intervention Triage Strategy
AF: Atrial Fibrillation
AMI: Acute myocardial infarction
CABG: Coronary artery bypass graft
CI: Confidence interval
HLOS: Hospital Length of Stay
ICD: International Classification of Diseases
ICU: Intensive Care Unit
INFORM: Informing Fresh Versus Old Red Cell Management
LOS: Length of Stay
OR: Odds ratio
pRBC: Packed red blood cell
QHAPDC: Queensland Health Admitted Patient Data Collection
RBC: Red blood cell
RCT: Randomised control trial
RECESS: Red-Cell Storage Duration Study
STROBE: Strengthening the Reporting of Observational Studies in Epidemiology

## References

[1] Daly DJ, et al. Anticoagulation, bleeding and blood transfusion practices in Australasian cardiac surgical practice. Anaesth Intensive Care. 2007;35:760–8. 17933164 10.1177/0310057X0703500516

[2] Kilic A, Whitman GJ. Blood transfusions in cardiac surgery: indications, risks, and conservation strategies. Ann Thorac Surg. 2014;97:726–34. 24359936 10.1016/j.athoracsur.2013.08.016

[3] Patel NN, Murphy GJ. Evidence-based Red Blood Cell Transfusion practices in Cardiac surgery. Transfus Med Rev. 2017;31:230–5. 28826760 10.1016/j.tmrv.2017.06.001

[4] Shah A, et al. Fresh versus old red cell transfusions: what have the recent clinical trials found? Curr Opin Hematol. 2016;23:550–6. 27518928 10.1097/MOH.0000000000000283

[5] Irving AH et al. Impact of patient blood management guidelines on blood transfusions and patient outcomes during cardiac surgery. J Thorac Cardiovasc Surg 2020;160: 437 – 45.e420. 10.1016/j.jtcvs.2019.08.102 31711621

[6] Carson JL, et al. JAMA. 2016;316:2025–35. 10.1001/jama.2016.9185 27732721

[7] Gerber DR. Risks of packed red blood cell transfusion in patients undergoing cardiac surgery. J Crit Care. 2012;27:e737731–9. 10.1016/j.jcrc.2012.05.007 22762927

[8] Koch CG, et al. Morbidity and mortality risk associated with red blood cell and blood-component transfusion in isolated coronary artery bypass grafting. Crit Care Med. 2006;34:1608–16. 16607235 10.1097/01.CCM.0000217920.48559.D8

[9] Stone GW, et al. Impact of major bleeding and blood transfusions after cardiac surgery: analysis from the Acute catheterization and urgent intervention triage strategY (ACUITY) trial. Am Heart J. 2012;163:522–9. 22424026 10.1016/j.ahj.2011.11.016

[10] Engoren M, et al. The Effect on Long-Term Survival of Erythrocyte Transfusion Given for Cardiac Valve Operations. Ann Thorac Surg. 2009;88:95–e100103. 19559202 10.1016/j.athoracsur.2009.04.047

[11] García-Roa M, et al. Red blood cell storage time and transfusion: current practice, concerns and future perspectives. Blood Transfus. 2017;15:222–31. 28518049 10.2450/2017.0345-16 PMC5448828

[12] Steiner ME, et al. Effects of Red-Cell Storage Duration on patients undergoing cardiac surgery. N Engl J Med. 2015;372:1419–29. 25853746 10.1056/NEJMoa1414219 PMC5442442

[13] McQuilten ZK, et al. Effect of age of red cells for transfusion on patient outcomes: a systematic review and meta-analysis. Transfus Med Rev. 2018;32:77–88. 29526337 10.1016/j.tmrv.2018.02.002

[14] Ng MSY, et al. Transfusion of packed red blood cells at the end of shelf life is associated with increased risk of mortality - a pooled patient data analysis of 16 observational trials. Haematologica. 2018;103:1542–8. 29794148 10.3324/haematol.2018.191932 PMC6119129

[15] Goel R, et al. Red blood cells stored 35 days or more are associated with adverse outcomes in high-risk patients. Transfusion. 2016;56:1690–8. 27062463 10.1111/trf.13559

[16] Queensland Health - Statewide Cardiac Clinical Network. (2020). Queensland Cardiac Outcomes Registry Annual Report 2020. Queensland: Queensland Health. https://clinicalexcellence.qld.gov.au/sites/default/files/docs/priority-area/clinical-engagement/networks/cardiac/qcor-annual-report-2020.pdf.

[17] McQuilten ZK. at al. Transfusion practice varies widely in cardiac surgery: Results from a national registry. J Thorac Cardiovasc Surg 2014;147:1684-90. 10.1016/j.jtcvs.2013.10.051 24332109

[18] van de Watering L, et al. Effects of storage time of red blood cell transfusions on the prognosis of coronary artery bypass graft patients. Transfusion. 2006;46:1712–8. 17002627 10.1111/j.1537-2995.2006.00958.x

[19] Yap C-H, et al. Age of Transfused Red cells and early outcomes after cardiac surgery. Ann Thorac Surg. 2008;86:554–9. 18640333 10.1016/j.athoracsur.2008.04.040

[20] McKenny M, et al. Age of transfused blood is not associated with increased postoperative adverse outcome after cardiac surgery. Br J Anaesth. 2011;106:643–9. 21414977 10.1093/bja/aer029

[21] Andreasen JJ, et al. Storage time of allogeneic red blood cells is associated with risk of severe postoperative infection after coronary artery bypass grafting☆. Eur J Cardiothorac Surg. 2011;39:329–34. 20702101 10.1016/j.ejcts.2010.06.019

[22] Kor DJ, et al. Red Blood Cell Storage Lesion. Bosn J Basic Med Sci. 2009;9(Suppl 1):S21–7. 19912115 10.17305/bjbms.2009.2750 PMC5655167

[23] Karon BS, et al. Changes in Band 3 oligomeric state precede cell membrane phospholipid loss during blood bank storage of red blood cells. Transfusion. 2009;49:1435–42. 19389033 10.1111/j.1537-2995.2009.02133.x PMC3649012

[24] D’Alessandro A, et al. Red blood cell storage: the story so far. Blood Transfus. 2010;8:82–8. 20383300 10.2450/2009.0122-09 PMC2851210

[25] Bennett-Guerrero E, et al. Evolution of adverse changes in stored RBCs. Proc Natl Acad Sci USA. 2007;104:17063–8. 17940021 10.1073/pnas.0708160104 PMC2040393

[26] Adams F et al. Biochemical storage lesions occurring in nonirradiated and irradiated red blood cells: a brief review. BioMed Res Int 2015;968302. 10.1155/2015/968302 PMC4325969 25710038

[27] Koster A, et al. Transfusion of 1 and 2 units of red blood cells does not increase mortality and organ failure in patients undergoing isolated coronary artery bypass grafting. Eur J Cardiothorac Surg. 2015;49:931–6. 26201957 10.1093/ejcts/ezv252

[28] Bhaskar B, et al. Impact of blood product transfusion on short and long-term survival after cardiac surgery: more evidence. Ann Thorac Surg. 2012;94:460–7. 22626751 10.1016/j.athoracsur.2012.04.005

[29] Leal-Noval SR, et al. Transfusion of Blood Components and postoperative infection in patients undergoing cardiac surgery(*). Chest. 2001;119:1461. 11348954 10.1378/chest.119.5.1461

[30] Banbury MK, et al. Transfusion increases the risk of postoperative infection after Cardiovascular surgery. J Am Coll Surg. 2006;202:131–8. 16377506 10.1016/j.jamcollsurg.2005.08.028

[31] Mariscalco G, et al. Red blood cell transfusion is a determinant of neurological complications after cardiac surgery. Interact Cardiovasc Thorac Surg. 2014;20:166–71. 25368133 10.1093/icvts/ivu360

[32] Maxwell MJ, Wilson MJA. Complications Blood Transfus Continuing Educ Anaesth Crit Care Pain. 2006;6:225–9.

[33] Gould S, et al. Packed red blood cell transfusion in the intensive care unit: limitations and consequences. Am J Crit Care. 2007;16:39–48. quiz 49. 17192525 10.4037/ajcc2007.16.1.39

[34] Horvath KA, et al. Blood transfusion and infection after cardiac surgery. Ann Thorac Surg. 2013;95:2194–201. 23647857 10.1016/j.athoracsur.2012.11.078 PMC3992887

[35] Bove T, et al. The incidence and risk of acute renal failure after cardiac surgery. J Cardiothorac Vasc Anesth. 2004;18:442–5. 15365924 10.1053/j.jvca.2004.05.021

