# Supplementary material for: Effects of red blood cell transfusion on patients undergoing cardiac surgery in Queensland – a retrospective cohort study
Source: J Cardiothorac Surg. 2024 Aug 1;19:475. doi: 10.1186/s13019-024-02974-7 (PMC11293042; doi:10.1186/s13019-024-02974-7)
Supplement: Supplementary file 1 — Supplementary Material 1 [file 13019_2024_2974_MOESM1_ESM.docx]

Supplementary table S1: STROBE Statement—Checklist of items that should be included in reports of ***cohort studies***

|  | | Item No | Recommendation | Page No |
| --- | --- | --- | --- | --- |
| **Title and abstract** | | 1 | (*a*) Indicate the study’s design with a commonly used term in the title or the abstract  *Effects of red blood cell transfusion on patients undergoing cardiac surgery in Queensland – a retrospective cohort study* |  |
|  |  |  | (*b*) Provide in the abstract an informative and balanced summary of what was done and what was found  *What was done:*  *Data from the Queensland Health Admitted Patient Data Collection database from 2007-2013 was retrospectively analysed. Patients undergoing coronary artery bypass graft and valvular repair were included. Multivariable logistic regression was used to examine the effect of red blood cell age (<35 days vs ≥35 days) on in-hospital mortality and morbidity. As a secondary analysis, outcomes associated with the number of red blood cell units transfused (≤4 units vs ≥5 units) were also assessed.*  *Main findings:*  *A total of 4514 patients undergoing cardiac surgery received packed red blood cell transfusion. Of these 292 (6.5%) received packed red blood cells ≥35 days. No difference in in-hospital mortality or frequency of complications was observed.*  *Transfusion of ≥5 units of pRBCs compared to the ≤4 units was associated with higher rates of in-hospital mortality (5.6% versus 1.3%), acute renal failure (17.6% vs 8%), infection (10% vs 3.4%), and acute myocardial infarction (9.2% vs 4.3%). On determining the odds ratio for said complications, infection carried an odds ratio of 1.37 between groups (CI=0.9-2.09; p=0.14) and stroke/neurological complications, 1.59 (CI=0.96-2.63; p=0.07).* |  |
| Introduction | | | | |
| Background/rationale | | 2 | Explain the scientific background and rationale for the investigation being reported  *Packed red blood cell (pRBC) transfusion is relatively safe and a mainstay in cardiac surgery. However, red blood cells undergo biochemical changes during storage and there is limited evidence on clinical effects of transfusing blood nearing end-of shelf life.* |  |
| Objectives | | 3 | State specific objectives, including any prespecified hypotheses  *This study aimed to evaluate the effects of blood transfusion on mortality and morbidity with pRBCs close to end of shelf-life (≥35 days) versus fresher blood on patients undergoing cardiac surgery through a multi-centre retrospective database analysis. As a parallel objective, transfusion-related adverse outcomes in relation to quantity of pRBCs transfused were characterised.* |  |
| Methods | | | | |
| Study design | | 4 | Present key elements of study design early in the paper  *A retrospective cohort study* |  |
| Setting | | 5 | Describe the setting, locations, and relevant dates, including periods of recruitment, exposure, follow-up, and data collection  *Queensland public hospitals between 2007 to 2013 (The Prince Charles Hospital, The Princess Alexandra Hospital, Gold Coast University Hospital, and Townsville University Hospital).* |  |
| Participants | | 6 | (*a*) Give the eligibility criteria, and the sources and methods of selection of participants. Describe methods of follow-up  *Patients were eligible for inclusion if they were 16 years or older and underwent CABG surgery or Valvular repair/replacement surgery (Figure 1).*  *Patients were excluded if they did not receive at least one unit of pRBC transfusion. Since the maximum allowed shelf-life of pRBCs in Australia is 42 days, any entries of pRBCs beyond this age were taken as errors in data entry and excluded from analysis.* |  |
|  |  |  | (*b*) For matched studies, give matching criteria and number of exposed and unexposed  *N/A* |  |
| Variables | | 7 | Clearly define all outcomes, exposures, predictors, potential confounders, and effect modifiers. Give diagnostic criteria, if applicable  *The primary patient groups compared were those patients receiving pRBCs with an average age of <35 days versus pRBCs with an average age ≥35 days. As a secondary analysis, patients receiving ≤4 units pRBCs were compared to those receiving ≥5 units*  *The primary outcome was in-hospital mortality. Secondary outcomes were the incidence of transfusion-associated complications. Complications of interest were infection, atrial fibrillation, other dysrhythmia, cardiac arrest/acute myocardial infarction, acute renal failure, and stroke/neurological complications.* |  |
| Data sources/ measurement | | 8* | For each variable of interest, give sources of data and details of methods of assessment (measurement). Describe comparability of assessment methods if there is more than one group  *Patient transfusion data is managed centrally on AUSLAB, the laboratory information system of Pathology Queensland, a state government service which catalogues every patient receiving blood product transfusions in a Queensland public hospital. Information associated with the transfusion such as blood product type, age of blood product and quantitative also collected.*  *AUSLAB data was crossmatched with the Queensland Health Admitted Patient Data Collection (QHAPDC) database, a state-wide data collection service capturing information on every patient admitted to a Queensland Health public hospital. Routinely collected information available from QHAPCE includes admission outcome, hospital length of stay, and attributed International Classification of Diseases (ICD) codes for primary and additional diagnoses.* |  |
| Bias | | 9 | Describe any efforts to address potential sources of bias  *Multivariate logistic regression* |  |
| Study size | | 10 | Explain how the study size was arrived at  *No formal study size calculation* |  |
| Quantitative variables | | 11 | Explain how quantitative variables were handled in the analyses. If applicable, describe which groupings were chosen and why  *Cohort characteristics and outcomes were first summarised descriptively, using medians with interquartile ranges for continuous variables, and frequencies with percentages for categorical variables. Continuous outcomes (i.e., hospital length of stay) were summarised using linear regression. Categorical outcomes (mortality and frequency complications) were summarised using multivariable logistic regression to determine odds ratio of incidence* |  |
| Statistical methods | | 12 | (*a*) Describe all statistical methods, including those used to control for confounding |  |
|  |  |  | (*b*) Describe any methods used to examine subgroups and interactions |  |
|  |  |  | (*c*) Explain how missing data were addressed |  |
|  |  |  | (*d*) If applicable, explain how loss to follow-up was addressed |  |
|  |  |  | (*e*) Describe any sensitivity analyses  *Outcomes (mortality and frequency complications) were summarised using multivariable logistic regression to determine odds ratio of incidence. Variables used as covariates in this analysis, for both outcomes of mortality and complication incidence, included*  *•Age, sex, year of transfusion, HLOS, surgery indication, number of blood products (RBC, plasma, platelet, cryoprecipitate, granulocyte), and incidence complications (Infection, AF, Other Dysrhythmia, Cardiac arrest/AMI, Acute renal failure, Stroke/neurological)*  *Statistical significance for mortality and complication incidence was defined as p<0.05.* |  |
| Results | | | |  |
| Participants | | 13* | (a) Report numbers of individuals at each stage of study—e.g., numbers potentially eligible, examined for eligibility, confirmed eligible, included in the study, completing follow-up, and analysed |  |
|  |  |  | (b) Give reasons for non-participation at each stage |  |
|  |  |  | 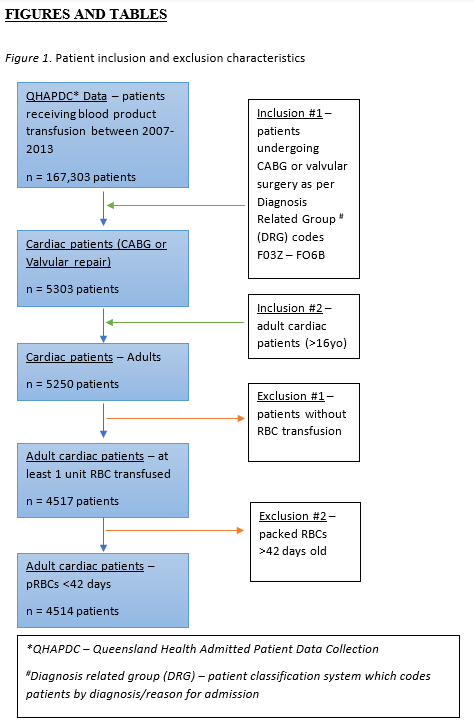(c) Consider use of a flow diagram |  |
| Descriptive data | | 14* | (a) Give characteristics of study participants (e.g., demographic, clinical, social) and information on exposures and potential confounders |  |
|  |  |  | (b) Indicate number of participants with missing data for each variable of interest |  |
|  |  |  | (c) Summarise follow-up time (e.g., average and total amount)  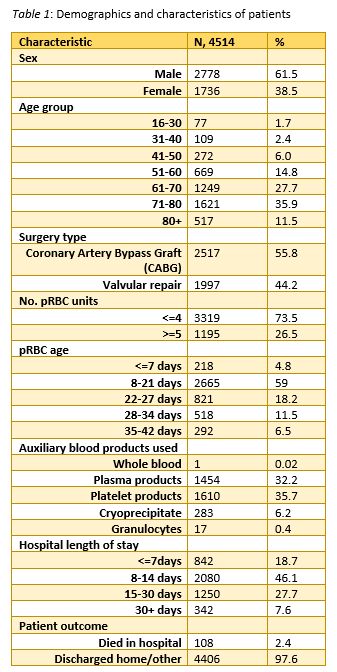 |  |
| Outcome data | | 15* | Report numbers of outcome events or summary measures over time  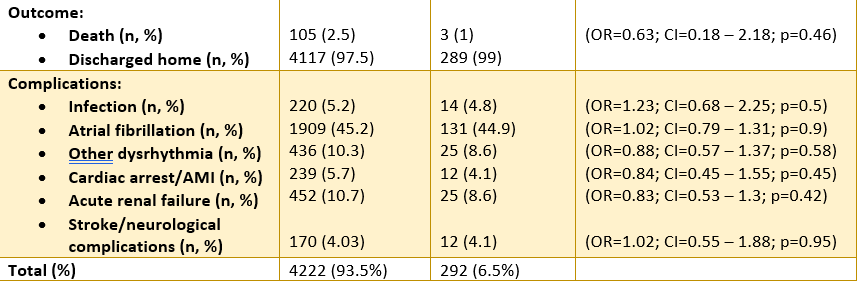AGE of pRBC <35 days >35 days |  |
| Main results | 16 | (*a*) Give unadjusted estimates and, if applicable, confounder-adjusted estimates and their precision (e.g., 95% confidence interval). Make clear which confounders were adjusted for and why they were included | |  |
|  |  | (*b*) Report category boundaries when continuous variables were categorized | |  |
|  |  | (*c*) If relevant, consider translating estimates of relative risk into absolute risk for a meaningful time period  *Of the total population, 4 222 (93.5%) patients received pRBCs with average age fresher than 35 days and 292 (6.5%) received pRBCs ≥35 days (Table 2). In-hospital mortality was 2.5% in the <35-day cohort and 1% in the ≥35-day cohort, though this result was not statistically significant (OR 0.63; CI=0.18 to– 2.18; p=0.46). No clinical or statistical differences in frequency of complications were observed between groups.*  *Comparing patients who received ≤4 units of pRBCs (n=3 319, 73.5%) to those who received ≥5 units (n=1195, 26.5%), Table 3, the in-hospital mortality was 5.6% and 1.3%, respectively (OR 1.08), though this result was statistically non-significant (CI=0.56 to 2.09; p=0.81). Hospital length of stay was respectively longer (15 days versus 10 days median; Estimate=6.02; CI=5.36 to 6.69; p=0.001)). The frequency of complications was higher for patients receiving ≥5 units of pRBCs including; acute renal failure (17.6% vs 8%), infection (10% vs 3.4%), and cardiac arrest/acute myocardial infarction (9.2% vs 4.3%); p<0.0011. Odds ratio for infection, comparing ≥5-unit to ≤4-unit groups, was 1.37 (CI=0.9 to 2.09; p=0.14) and 1.59 for stroke/neurological complications (CI=0.96 to 2.63; p=0.07).* | |  |
| Other analyses | 17 | Report other analyses done—e.g., analyses of subgroups and interactions, and sensitivity analyses  *N/A* | |  |
| Discussion | | | | |
| Key results | 18 | Summarise key results with reference to study objectives  *In summary, it was found that transfusing pRBCs close to end of shelf-life (≥35 days) in cardiac surgery patients was not associated with increased early morbidity or mortality compared to patients receiving blood fresher than 35 days (Table 2). On comparing patients receiving ≤4 vs ≥5 units of pRBC, higher rates of mortality and complications (specifically infection, acute renal failure and cardiac arrest/AMI) were observed in the ≥5 units group, though statistical significance in these results were not achieved.* | |  |
| Limitations | 19 | Discuss limitations of the study, taking into account sources of potential bias or imprecision. Discuss both direction and magnitude of any potential bias  *Firstly, as a retrospective observational study, temporality is removed from the associations studied - it is unclear whether less quantity of pRBCs resulted in healthier patients or healthier patients resulted in less pRBCs units used. Also, the data obtained through AUSLAB and QHAPDC registries, permitted maximal completeness of data and minimised data collection biases in sacrifice for detail – for example, data including patient comorbidities, haemoglobin levels, anticoagulation; units transfused in preoperative versus intraoperative/postoperative period, cardio-pulmonary bypass time; and specific outcome measures such as cause of death and ICU ventilation time/LOS.* | |  |
| Interpretation | 20 | Give a cautious overall interpretation of results considering objectives, limitations, multiplicity of analyses, results from similar studies, and other relevant evidence  *Transfusion of pRBCs closer to end of shelf-life was not shown to be associated with increased mortality or morbidity in cardiac surgical patients*. | |  |
| Generalisability | 21 | Discuss the generalisability (external validity) of the study results  *Increased generalisability was obtained by analysing a multicentre population across all public cardiothoracic centres in Queensland.* | |  |
| Other information | | | | |
| Funding | 22 | Give the source of funding and the role of the funders for the present study and, if applicable, for the original study on which the present article is based  *Professor John F. Fraser received grant funding from The Prince Charles Hospital Foundation (MS2014-50) that has supported this project. Australian Governments fund Australian Red Cross Lifeblood for the provision of blood, blood products and services to the Australian Community.* | |  |

Supplementary table S2: International Classification of Diseases (ICD) codes used to identify transfusion-related adverse events

| Adverse event | ICD-codes |
| --- | --- |
| Atrial fibrillation | |
|  | I48 |
|  | I480 |
|  | I481 |
|  | I483 |
|  | I484 |
|  | I489 |
| Other Dysrhythmia | |
|  | I470 |
|  | I471 |
|  | I472 |
|  | I479 |
|  | I47 |
|  | I490 |
|  | I491 |
|  | I493 |
|  | I494 |
|  | I495 |
|  | I498 |
|  | I499 |
|  | I49 |
| Infection | |
|  | T814 |
|  | T857 |
|  | T826 |
|  | T827 |
|  | J985 |
|  | J85 |
|  | J853 |
|  | I301 |
|  | I38 |
|  | I33 |
|  | I330 |
|  | 1400 |
|  | A400 |
|  | A402 |
|  | A403 |
|  | A408 |
|  | A409 |
|  | A410 |
|  | A411 |
|  | A412 |
|  | A414 |
|  | A4150 |
|  | A4151 |
|  | A4152 |
|  | A4158 |
|  | A418 |
|  | A419 |
|  | B377 |
|  | A021 |
|  | A327 |
|  | A401 |
|  | A413 |
|  | O85 |
|  | J12 |
|  | J120 |
|  | J121 |
|  | J122 |
|  | J123 |
|  | J128 |
|  | J129 |
|  | J13 |
|  | J14 |
|  | J15 |
|  | J150 |
|  | J151 |
|  | J152 |
|  | J153 |
|  | J154 |
|  | J155 |
|  | J156 |
|  | J157 |
|  | J158 |
|  | J159 |
|  | J16 |
|  | J160 |
|  | J168 |
|  | J17 |
|  | J170 |
|  | J171 |
|  | J172 |
|  | J173 |
|  | J178 |
|  | J18 |
|  | J180 |
|  | J181 |
|  | J182 |
|  | J188 |
|  | J189 |
|  | J851 |
| Acute Renal Failure | |
|  | N990 |
|  | N170 |
|  | N171 |
|  | N172 |
|  | N178 |
|  | N179 |
|  | N17 |
| Cardiac arrest/AMI | |
|  | I460 |
|  | I461 |
|  | I469 |
|  | I46 |
|  | I210 |
|  | I211 |
|  | I212 |
|  | I213 |
|  | I214 |
|  | I219 |
|  | I21 |
| Stroke/neurological complications | |
|  | I600 |
|  | I601 |
|  | I602 |
|  | I603 |
|  | I604 |
|  | I605 |
|  | I606 |
|  | I607 |
|  | I608 |
|  | I609 |
|  | I610 |
|  | I611 |
|  | I612 |
|  | I613 |
|  | I614 |
|  | I615 |
|  | I616 |
|  | I617 |
|  | I618 |
|  | I619 |
|  | I629 |
|  | I630 |
|  | I631 |
|  | I632 |
|  | I633 |
|  | I634 |
|  | I635 |
|  | I636 |
|  | I637 |
|  | I638 |
|  | I639 |
|  | I64 |
|  | I650 |
|  | I651 |
|  | I652 |
|  | I653 |
|  | I658 |
|  | I659 |
|  | I661 |
|  | I66 |
|  | I662 |
|  | I663 |
|  | I664 |
|  | I660 |
|  | I668 |
|  | I669 |
|  | I65 |
